# Cattle-related risk factors for malaria in southwest Ethiopia: a cross-sectional study

**DOI:** 10.1186/s12936-022-04202-w

**Published:** 2022-06-10

**Authors:** Kallista Chan, Jorge Cano, Fekadu Massebo, Louisa A. Messenger

**Affiliations:** 1 Department of Disease Control, Faculty of Infectious and Tropical Diseases, London School of Hygiene & Tropical Medicine, London, UK; 2 Expanded Special Project for Elimination of NTDs, World Health Organization Regional Office for Africa, Brazzaville, Republic of Congo; 3 Department of Biology, Collage of Natural Sciences, Arba Minch University, Arba Minch, Ethiopia

**Keywords:** *Anopheles*, Cattle, Ethiopia, Livestock, Malaria, Vector control

## Abstract

**Background:**

Despite the low to moderate intensity of malaria transmission present in Ethiopia, malaria is still a leading public health problem. Current vector control interventions, principally long-lasting insecticidal nets and indoor residual spraying, when deployed alone or in combination, are insufficient to control the dominant vector species due to their exophagic and exophilic tendencies. Zooprophylaxis presents a potential supplementary vector control method for malaria; however, supporting evidence for its efficacy has been mixed.

**Methods:**

To identify risk factors of malaria and to estimate the association between cattle and *Anopheles* vector abundance as well as malaria risk, a cross-sectional study was conducted in a village near Arba Minch, Ethiopia. Epidemiological surveys (households = 95, individuals = 463), mosquito collections using CDC light traps and a census of cattle and human populations were conducted. To capture environmental conditions, land cover and water bodies were mapped using satellite imagery. Risk factor analyses were performed through logistic, Poisson, negative binomial, and spatial weighted regression models.

**Results:**

The only risk factor associated with self-reported malaria illness at an individual level was being a child aged 5 or under, where they had three times higher odds than adults. At the household level, variables associated with malaria vector abundance, especially those indoors, included socioeconomic status, the proportion of children in a household and cattle population density.

**Conclusions:**

Study results are limited by the low abundance of malaria vectors found and use of self-reported malaria incidence. Environmental factors together with a household’s socioeconomic status and host availability played important roles in the risk of malaria infection in southwest Ethiopia. Cattle abundance in the form of higher cattle to human ratios may act as a protective factor against mosquito infestation and malaria risk. Humans should remain indoors to maximize potential protection against vectors and cattle kept outside of homes.

**Supplementary information:**

The online version contains supplementary material available at 10.1186/s12936-022-04202-w.

## Background

Of all vector-borne diseases, malaria is the most lethal; resulting in an annual mortality of over 400,000 deaths [1]. Its burden is the heaviest in sub-Saharan Africa, where there is holoendemic transmission. However, Ethiopia lies on the belt fringing the Sahel and, unlike its southern neighbours, is considered to have low to moderate intensity of malaria transmission, due to its variable climate and higher elevations [2]. Nonetheless, malaria is one of Ethiopia’s leading public health problems, creating the highest proportion of outpatient morbidity [3]. Moreover, due to its seasonality, unstable transmission, and low levels of immunity amongst individuals in Ethiopia, the country is highly prone to epidemics [4, 5]. It has been estimated that 75% of total land area and 60% of the population are at risk [3]. *Plasmodium falciparum* and *Plasmodium vivax* are the two major aetiological agents responsible, accounting for 60% and 40% of cases, respectively. In terms of vector species, *Anopheles arabiensis*, which belongs to the *Anopheles gambiae* species complex, predominates, followed by *Anopheles pharoensis* [6]. Both vectors have wide preferences in resting and feeding behaviours, but they tend to be more exophilic, exophagic and zoophilic [7, 8]. *Anopheles stephensi*, originally from India, has also recently established in Ethiopia and has zoophagic tendencies as well [9].

Amongst others, the current strategies adopted by the National Malaria Elimination Programme (NMEP) in Ethiopia have emphasized improving vector control, of which insufficient coverage remains a large problem [3]. In 2015, the proportion of at-risk households that were protected by at least one long-lasting insecticidal net (LLIN) or round of indoor residual spraying (IRS) had reached 71%, which, for an area of low to moderate malaria endemicity, is adequate [10]. Bed-net utilization was however lower, where only 45% of children under 5 reportedly slept under a net the previous night [10]. In terms of IRS, less than 30% of targeted areas have received spraying in the past 12 months [10]. Although coverage of both interventions needs to increase, rising levels of insecticide resistance have also been reported in malaria vectors across the country [3, 6, 11]. Moreover, LLINs and IRS both primarily target indoor feeding and resting mosquitoes and may not be as effective against Ethiopia’s major vector species; additional non-insecticidal-based interventions are required to tackle the exophagic and exophilic behaviours of these vectors. One such alternative is zooprophylaxis.

Zooprophylaxis is the use of cattle or other livestock to divert mosquitoes from biting humans, provided that these vectors are opportunistic feeders and do not have a high, or exclusive, preference for particular hosts. In theory, it is a highly potent control method, due to its capacity to decrease malaria transmission to humans and prevent further parasite reproduction, by having mosquitoes feed on dead-end hosts. Historically, zooprophylaxis has been recognized and recommended as a control measure against many mosquito-borne diseases by the World Health Organization (WHO) [12]. In practice, however, there is contradicting evidence to support its efficacy. It has been debated whether the use of cattle actually results in the polar effect of zooprophylaxis-zoopotentiation: where livestock attract vectors towards humans by creating additional bloodmeal sources or breeding habitats (i.e. cattle hoofprints) and increase malaria risk [13]. Previous studies have found either effect to arise in specific situations but a key factor that influences which occurs is the feeding behaviour of the predominant vector species; proclivity to feed on domestic animals over humans can lead to zooprophylaxis [14, 15].

Since Ethiopia’s dominant and emerging malaria vectors are primarily zoophilic, it has been speculated that zooprophylaxis has considerable potential as a secondary vector control method. Accordingly, multiple studies related to cattle and their protective effect against malaria have been conducted in Ethiopia [6, 16–22]. Whilst some results of these studies suggested that the use of cattle can divert malaria vectors away from humans, others did not support any effect and even provided evidence of zoopotentiation [16, 23, 24]. With this disparity in results, it is unclear whether zooprophylaxis can be used as a supplementary vector control strategy. Moreover, relative to other countries in Eastern Africa, Ethiopia has high livestock density (and is a major exporter of livestock), which may inevitably increase vector population sizes. There is a need for more in-depth information (such as cattle ownership, cattle-keeping practices and livestock density distribution) to properly distinguish the conditions that give rise to these two contrasting effects. This study aimed to identify socio-environmental risk factors of malaria and to estimate the associations between fine-scale cattle-related factors and *Anopheles* vector abundance as well as malaria risk in southwest Ethiopia.

## Methods

### Study area and sample selection

This study was undertaken in July 2017 in Shelle Mella village, 18 km south of Arba Minch town, in southwest Ethiopia. Shelle Mella is located at 5.86639° N, 37.47583° E, at a constant altitude of 1200 m asl next to Lake Chamo (Additional file 1: Fig. S1). There are relatively stable sub-humid climate conditions throughout the year, with the main and secondary wet seasons in April–May and September–October, respectively. During the study period, the average monthly rainfall was 63 mm and the average minimum and maximum monthly temperatures were 17 and 26 °C. The inhabitants were subsistence farmers where their main source of income were cash crops, such as bananas and mangoes. Housing in the village was diverse; wall surfaces were rough, smooth, or painted mud walls, whereas roofs were thatched or metal. Indoor residual spraying using bendiocarb (carbamate) was performed in July and August 2016, whereas deltamethrin-treated LLINs were distributed in January 2017.

The approximate locations of each compound (a single-family household structure) in the village were mapped and exported to ArcGIS v10.5 for household sample selection [25]. To maintain spatial heterogeneity, households closest to cell centroids of 150 by 150 m grids were chosen for entomological and epidemiological surveys.

### Mosquito collection and processing

Indoor and outdoor sampling for adult female *Anopheles* mosquitoes was carried out in 95 surveyed households using CDC light traps across 34 nights; an average of 14 households was sampled per week. Each household was only sampled once, and indoor and outdoor collections were conducted simultaneously. Indoors, traps were hung 45 cm above the floor at the feet of sleeping persons protected by untreated nets. Outdoors, traps were placed 150 cm above the ground near the cattle inside the compound or inside the cattle shed. If the household did not own any cattle, traps were placed no more than 10 m away from the house. Light traps were switched on at 18:00 and off at 06:00 the following morning. Collected mosquitoes, distinguished by indoor or outdoor collections, were then transported to the entomology laboratory at Arba Minch University (AMU) for processing. They were sorted and identified to species complex level, with a dissecting microscope, according to the morphological key by Gillies and Coetzee [26].

### Epidemiological survey and census

A modified version of the Malaria Indicator Survey Household Questionnaire was developed to encapsulate more detail on cattle-keeping practices (CKP). It was administered through face-to-face interviews with heads of households of the same 95 entomologically surveyed households. The following data were collected: household location, size and construction materials, socio-demographic information, ownership of assets, indoor- and outdoor-sleeping conditions and information on malaria interventions. The period prevalence of self-reported malaria episodes in the last 30 days was also collected.

A census of all cattle and humans in Shelle Mella was conducted in order to estimate the availability of animal and human hosts. The approximate night-time locations of both cattle and humans were geo-referenced using a handheld Global Positioning System 60™ (Garmin International Inc, USA). Information on the types of CKP, categorized into use of cattle sheds or cattle in open conditions within the compound, adopted by households was also collected. Cattle and human population density, and the density of households for each CKP were estimated using a Kernel Density Estimator and fed into a spatial raster of 15 × 15 m resolution.

### Statistical analysis

#### Principal component analysis

To construct a relative index of socioeconomic status (SES), a principal component analysis (PCA) was conducted in STATA version 15.0 to combine household-level information on assets and housing quality. Three variables (ownership of a car and landline telephone and the type of fuel used) exhibited low variation across households and were excluded from the analysis. The 26 variables that were incorporated into the wealth index included: household size, assets pertaining to quality of the house (n = 7) and asset ownership by any member of the household (n = 18). Wealth indices were used to group households into quartiles according to their SES (low to high).

#### Mapping environmental variables

To map environmental variables such as land cover and water bodies in the study area, a Landsat 8 Operational Land Imager level-1 product measuring Earth’s surface radiation (in 9 spectral bands—from visible to thermal-infrared) was downloaded from the U.S. Geological Survey website [27]. Pan-sharpening processes and atmospheric corrections were applied to multispectral bands to obtain the Normalized Difference Vegetation Index (a measure of vegetation greenness) and Modified Normalized Difference Water Index (a measure of water saturation) (Additional file 1: Fig. S2). Potential mosquito vector breeding sites were derived by identifying water bodies through an interactive supervised classification of a false colour composite image formed with bands 5 (Near Infrared), 6 (Shortwave Infrared) and 4 (Red Visible). To quantify the distances of households to these potential breeding sites, Euclidean distances were calculated. All image classification and geoprocessing were conducted using ArcGIS 10.5 and *RSToolbox* package in R [25, 28].

#### Exploratory and risk factor analysis

A simple spatial exploratory analysis was conducted on the reports of malaria incidence and collected mosquitoes using inverse distance weighting (IDW). The technique estimates the values of spatial phenomena at non-sampled locations using values where observations exist, and accounting for the distance existing between locations where the outcome was measured. Further details on the implementation of this spatial analytic method are provided in Additional file 1: Text S1 and Fig. S3).

Risk factors included in all regression analyses were household-specific data collected from the epidemiological surveys, human and cattle population density collected from the census and environmental variables quantified by remote sensing data. All potential risk factors were checked for multi-collinearity using correlation matrices and variance inflation factors (VIF).

This study aimed to identify risk factors of malaria infection and to estimate the associations between fine-scale cattle-related factors and malaria risk as well as *Anopheles* vector abundance. To eliminate redundancy, explanatory variables that exceeded a correlation coefficient of 0.75 and/or a VIF of 10 were removed from candidate models.

To estimate the odds ratios (ORs) of whether an individual self-reportedly had malaria in the last 30 days, a univariate analysis of all variables was conducted using multi-level logistic regression that was adjusted to account for within-household clustering of cases. ORs were also adjusted for age, sex and SES. 

To estimate the associations between cattle-related factors and household malaria incidence, all variables showing evidence for a possible association (p < 0.15) were included in a Poisson regression analysis. The same procedure was adopted to estimate the associations between cattle-related factors and household malaria vector abundance (including indoors and outdoors), but negative binomial regression analyses were used. Whilst including potential confounders as covariates, model selection was then based on a combination of automatic stepwise procedures, model performance parameters and two indicators of goodness of fit, the Akaike Information Criterion (AIC) and the Bayesian Information Criterion (BIC). Since conventional regression analyses tend to overlook any potential spatial dependency, a test for spatial autocorrelation using Moran’s *I* was conducted. Where appropriate, geographically weighted regression (GWR) models were applied to account for spatial autocorrelation. All regression analyses were performed in R version 3.3.1 [29].

## Results

### Malaria vector composition, malaria incidence, and cattle density

Across 34 nights, 156 female mosquitoes of 6 different *Anopheles* species were collected from 95 households in the study village (Table 1). Of the 6 species, only *An. gambiae sensu lato* (*s.l*.), *Anopheles funestus s.l*. and *An. pharoensis* were known malaria vectors. *Anopheles gambiae s.l*. was the most abundant (83.3%). Generally, the mean number of malaria vectors found per household was greater indoors than outdoors (paired Wilcoxon test p = 0.024). When households were divided into those with (n = 71) and without cattle (n = 24), there were no significant differences between mean numbers of malaria vectors found per household inside or outside households with or without cattle (inside: p = 0.271, outside: p = 0.233). Around half (45.5%) of the *Anopheles* mosquitoes collected were blood-fed.

**Table 1:** The distribution of female *Anopheles* mosquitoes collected from households, both indoors and outdoors, and the proportions of blood-fed mosquitoes

Anopheles species	Households with cattle (n = 71)		Households without cattle (n = 24)		No. blood-fed (%)	Total (%)
	Indoors	Outdoors	Indoors	Outdoors		
*Anopheles gambiae* s.l.	52	23	33	22	58 (44.6)	130 (83.3)
*Anopheles garnhami*	2	3	1	2	4 (50.0)	8 (5.1)
*Anopheles tenebrosus*	3	2	2	0	3 (100.0)	7 (4.5)
*Anopheles funestus* s.l.	3	2	0	0	2 (40.0)	5 (3.2)
*Anopheles marshalli*	0	3	0	0	3 (100.0)	3 (1.9)
*Anopheles pharoensis*	1	2	0	0	1 (33.3)	3 (1.9)
Total	61 (39.1)	35 (22.4)	36 (23.1)	24 (15.4)	71 (45.5)	156 (100.0)

Amongst the 95 households that were inhabited by 463 people, 37 households had at least one person with self-reported malaria in the last 30 days; the total number of self-reported malaria cases was 49. Each sampled household had an average of 3 cattle. The village population census tallied a total of 6303 individuals and 2880 cattle in 1378 households.

### Spatial exploratory analysis

**Fig. 1:**
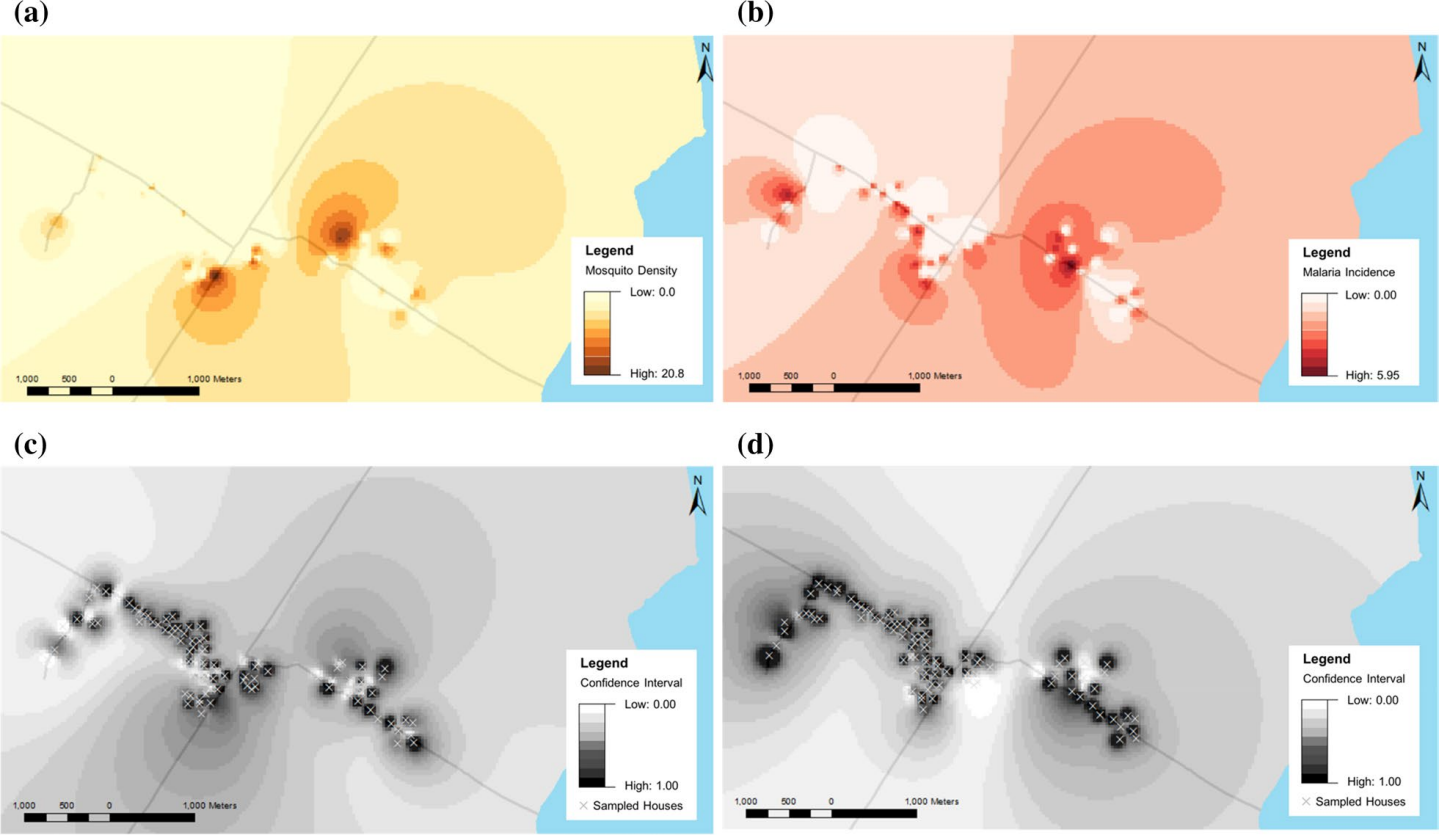
The predicted **A** mosquito density and **B** number of malaria incident cases per household across Shelle Mella, accompanied by the confidence intervals of the estimated numbers of **C** total mosquitoes collected and **D** malaria cases as estimates of the accuracy of the interpolations

Higher numbers of mosquitoes were found in the eastern side of the village (Fig. 1A). In contrast, mosquitoes were largely absent in households located in the western sub-villages. This general pattern was also predicted for indoor and outdoor captures (Additional file 1: Fig. S4A, B). Through cross validation, it was determined that about 10 to 15% of variance was explained by IDW interpolation of total and indoor mosquito density (Additional file 1: Table S2). However, when considering the predicted densities of mosquitoes found outdoors, higher root-mean-square error (RMSEs) were found in the predicted values across the non-sampled locations than that of the observed values, indicating the interpolated surface’s lack of predictive value.

Similar to predicted mosquito abundance across the village, higher malaria incidence was found in households east of the Arba Minch-Konso road, close to Lake Chamo (Fig. 1B). However, an area of low malaria incidence was also found in households closest to the lake. In the western part of the village, lower malaria incidence was found in households, except for those bordering the village, which had more malaria cases.

### Individual-level risk factor analysis

Children aged 5 years and below had almost three times higher odds of having malaria (aOR 2.88, 95% CI 1.17–7.11, p = 0.022; Additional file 1: Table S3). None of the other factors, including SES, prior IRS application and the number of nets owned, were associated with malaria incidence.

### Household-level risk factor analysis

There was no association between malaria risk and cattle population density at the household level (Table 2). However, fewer malaria vectors were associated with households with high cattle population density (p < 0.001) but also higher SES (p = 0.035). Specifically, fewer indoor malaria vectors were associated with households with high cattle population density (p = 0.003), high SES (p = 0.036) and lower proportions of children (p = 0.042). The number of outdoor mosquitoes, which was spatially clustered, was only determined by cattle population density.

**Table 2:** Factors associated with mosquito density (overall, indoors and outdoors) and malaria incidence within households (n = 95), whilst controlling for SES, IRS application and number of nets owned

Explanatory variable	Count	Standard error	Z-value	p-value	Moran’s I
Overall mosquito density					
Constant	0.830	0.411	2.268	0.023	0.00150 (p = 0.246)
Cattle population density	− 313.138	72.922	− 3.673	< 0.001	
SES	− 0.155	0.074	− 2.105	0.035	
IRS	− 0.031	0.375	− 0.082	0.935	
Number of nets	− 0.186	0.149	− 1.244	0.214	
Indoor mosquito density					
Constant	− 0.526	0.653	− 0.806	0.420	− 0.0243 (p = 0.743)
Cattle population density	− 250.391	84.179	− 2.975	0.003	
Proportion of children in household	1.888	0.930	2.030	0.042	
CKP: cattle in sheds	− 0.924	0.621	− 1.489	0.136	
CKP: cattle within compound	0.264	0.430	0.613	0.540	
SES	− 0.183	0.087	− 2.093	0.036	
IRS	− 0.275	0.436	− 0.631	0.528	
Number of nets	− 0.362	0.186	− 1.946	0.052	
Outdoor mosquito density*					
Constant	0.718	0.594	1.205	0.228	0.0295 (p = 0.035)
Cattle population density	− 131.252	75.268	− 1.744	0.081	
SES	− 0.066	0.082	− 0.805	0.421	
IRS	0.437	0.468	0.933	0.351	
Number of nets	0.054	0.188	0.289	0.772	
Number of reported malaria cases					
Constant	0.177	0.357	0.496	0.620	0.00687 (p = 0.190)
Cattle population density	− 133.805	70.939	− 1.886	0.059	
SES	− 0.036	0.063	− 0.571	0.568	
IRS	− 0.314	0.332	− 0.947	0.344	
Number of nets	− 0.169	0.152	− 1.110	0.267	

*Adjusted for spatial correlation using GWR spatial lag model

## Discussion

In this study village in southwest Ethiopia, the most abundant malaria vectors were *An. gambiae s.l*., followed by *An. funestus s.l*. and *An. pharoensis*, of which most were found indoors than outdoors. Households with fewer neighbouring cattle and of lower SES had higher numbers of malaria vectors. More malaria vectors were found inside households with higher proportions of children. The self-reported malaria incidence was 105 cases per 1000 person-month. At the household level, there was no strong association between malaria risk and any of the other cattle-related risk factors (number of cattle owned, cattle-to-human ratio and CKP). At the individual level, the only factor associated with greater malaria risk was children aged under 5, which had almost three times greater odds of disease.

Despite the region’s dominant vectors being primarily exophagic/exophilic, outdoor collections yielded lower malaria vector abundance than indoor collections [6]. This could be explained by two circumstances: either (1) the vectors’ behaviours are much more plastic than previously thought or (2) the CDC light traps were unintentionally trapping mosquitoes that had fed outdoors but went indoors to rest. The latter may also explain the fact that although CDC light traps tend to target host-seeking mosquitoes, around half the vectors that were captured were blood-fed. This occurrence should have been confirmed by bloodmeal identification, but this was not logistically feasible at the time of this study. Regardless, malaria vector abundances should be interpreted with caution due to their low densities collected in this study.

Households of lower SES, and hence lower quality of housing, were associated with higher mosquito densities, particularly those indoors. This is consistent with the wider literature: housing plays an important role in the degree of human mosquito contact as the presence of open eaves, cracks in walls, and roofs of poorly constructed houses increases the number of entry points for host-seeking vectors [30, 31]. This relationship was not consistent with malaria incidence, but could be attributed to the method used to construct the wealth index of households; using material assets as a proxy for SES have repeatedly failed to establish a significant relationship with malaria risk [32].

Of all cattle-related factors, only cattle population density around a household was associated with any of the four outcomes of interest. Specifically, fewer mosquitoes were found (indoors) when cattle densities around households were high. This suggests that the “community effect” of cattle was more important than household-level effects such as the number of cattle owned or CKP, both of which are more commonly measured in the literature (Additional file 1: Table S4) [16, 18, 20]. However, it must be considered that the abundance of malaria vectors was very low and could therefore have limited the ability to detect relevant covariates. The proportion of children inside a household was also positively associated with the number of malaria vectors collected indoors, which could be explained by low ITN use amongst children [33]. Hence some level of zooprophylaxis may be occurring; while *Anopheles* vectors were more attracted to human odour than cattle, they possessed more opportunistic and outdoor feeding tendencies and thus were diverted by the large cattle host availability outdoors [22]. This illustrates two important elements, although it did not translate to reduced malaria risk, (1) high cattle density around a household still provides some protection, especially against mosquitoes from entering indoors to feed and thus (2) humans should remain indoors to maximize protection against vectors. Fortunately, during the study period, community members were not observed sleeping outside.

Unlike other country- and zone-wide studies on malaria risk factors, this study found that, even during the dry season, children under 5 were at greater risk [34–36]. This observation, along with reported malaria incidence, implies that the study area actually harbours more stable transmission, since this pattern of increased risk in younger individuals reflects the age-dependence of malaria immunity that builds as a consequence of repeated infections over time [37, 38]. These findings concur with a study conducted less than 40 km away from Shelle Mella village, which also found that children aged below 5 had around 3 times the odds of malaria risk [33]. This suggests that the generalization of “unstable” malaria transmission across Ethiopia may be inaccurate and more fine-scale classification of areas by transmission intensity is needed to control malaria.

There are several limitations to this cross-sectional study. First, it was performed during the dry season and at a small spatial scale, with a small sample size. With such a small sample size, where only one person per household was interviewed and only one round of mosquito catches conducted per household, the study may not have had enough statistical power. In fact, low malaria vector abundance was observed, due not only to collections being made in the dry season but also potentially due to the choice of sampling trap (i.e. CDC light traps tend to capture host-seeking mosquitoes), and because ITN distribution had occurred six or seven months before mosquito collections. It is, therefore, recommended to conduct multiple cross-sectional surveys throughout a year instead, to account for seasonality which is an important characteristic of Ethiopia’s malaria transmission, and to use a variety of mosquito sampling techniques (including pit traps and spray catches to capture resting mosquitoes). Second, the quality of our secondary outcome, malaria incidence, was low as it was based on self-reporting and is hence subjected to information bias. Malaria incidence in this study could have been an over-estimation; in southwest Ethiopia, community members often defined malaria illness in terms of chills, fever and/or headaches [39].

Third, ground-based sampling of potential breeding sites, instead of reliance on remote sensing data, would have been more informative in determining mosquito abundance since wetlands are not necessarily the only source of vector breeding. In this case, it could only be speculated that during the dry season, lake edges accumulated small, temporary breeding sites suitable for *An. arabiensis*. This brings us to our fourth point: due to logistical constraints, vectors were not identified to species level by molecular methods and whilst *An. arabiensis* predominates this area of Ethiopia, it is not certain which species from the *An. funestus* complex were collected. Fifth, sporozoite rates and blood meal analyses were not conducted. As indicators of malaria transmission and feeding preferences, both factors would have contributed to investigating the protective factor of cattle. Finally, this cross-sectional study only collected a static record of cattle population density and household CKP. Further information on cattle (and human) movement, particularly at night, could have been collected using GPS trackers to determine average cattle distance of owned cattle from households and thus help disentangle the complexity behind these spatial vector-host relationships. Nevertheless, although this study was not able to infer a direct causal relationship of zooprophylaxis and reduction of malaria risk, it has identified that cattle population density should be examined in more detail in future studies. It would be highly useful to incorporate a component of cattle population density (perhaps CKP) in future Malaria Indicator Surveys.

## Conclusions

This study was limited by its small sample size, low malaria vector abundance and self-reporting malaria incidence. Nevertheless, in this region of southwest Ethiopia, cattle population density, rather than cattle ownership or CKP, provided some level of protection against malaria vectors from entering indoors. Thus, to ensure maximum protection against potential vectors, community members must  stay indoors at night. This factor, alongside distance of cattle from households, should be accounted for in future studies to unravel the true effects of cattle distribution on *Anopheles* mosquito density.

## Supplementary Information

**Additional file 1.** Study supplementary information.

## Data Availability

Not applicable.

## Abbreviations

AIC: Akaike Information Criterion
AMU: Arba Minch University
ASL: Above sea level
BIC: Bayesian Information Criterion
CDC: Centers for Disease Control and Prevention
CI: Confidence interval
CKP: Cattle-keeping practice
GLM: Generalized linear model
GWR: Geographically weighted regression
HBR: Human biting rate
HLC: Human landing catch
IRC: Indoor resting collection
IRS: Indoor residual spraying
LLIN: Long-lasting insecticidal net
LSHTM: London School of Hygiene and Tropical Medicine
OR: Odds ratio
PCA: Principal component analysis
SES: Socioeconomic status
VIF: Variance inflation factor
WHO: World Health Organization
