# Supplementary material for: Cattle-related risk factors for malaria in southwest Ethiopia: a cross-sectional study
Source: Malar J. 2022 Jun 10;21:179. doi: 10.1186/s12936-022-04202-w (PMC9188194; doi:10.1186/s12936-022-04202-w)
Supplement: Supplementary file 1 — Additional file 1. Study supplementary information. [file 12936_2022_4202_MOESM1_ESM.docx]

**Risk factors of malaria in southwest Ethiopia: a cross-sectional study**

Kallista Chan, Jorge Cano, Fekadu Massebo, Louisa A. Messenger


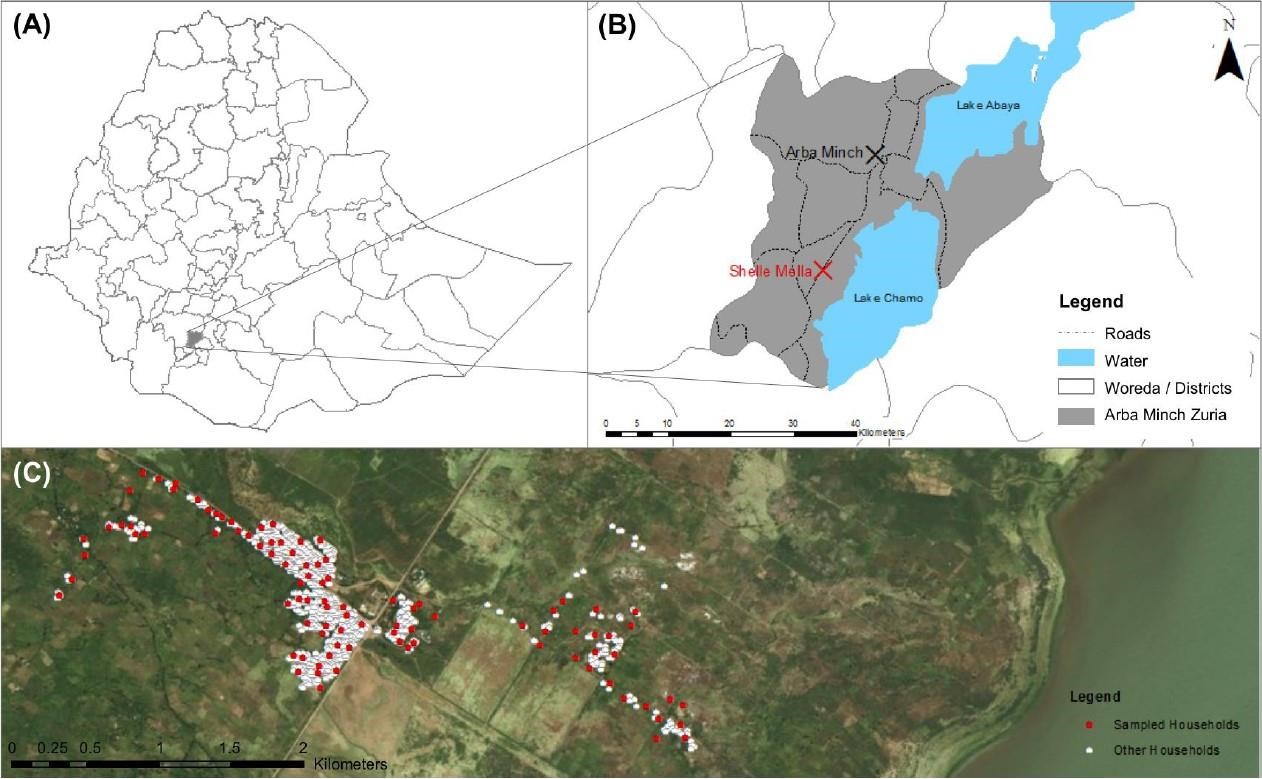


**Figure S1. (A)** Geographical location of the district (Arba Minch Zuria) in which the study was conducted relative to Ethiopia and **(B)** the location of the study village, Shelle Mella, in relation to Arba Minch town and the rift valley lakes.


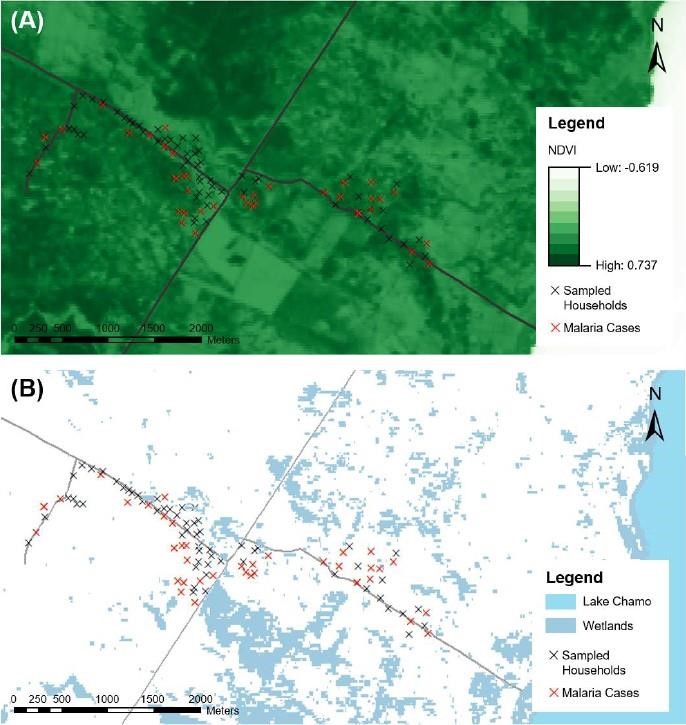


**Figure S2**. The **(A)** normalised difference vegetation index (NDVI) **(B)** water bodies, sorted into Lake Chamo and wetland areas, across the study village, Shelle Mella, extracted from a Landsat 8 Operational Land Imager.

**Text 1**. Spatial exploratory analysis.

The identification of clusters of malaria incidence and mosquito density within the study area was conducted using inverse distance weighting (IDW). The technique estimates the values of spatial phenomena at non-sampled locations using values where observations exist (Supplementary Figure 3); their mean values are calculated from neighbouring weighted locations. The weights are proportional to the proximity of sampled points to the non-sampled locations and can be specified by the IDW power coefficient; the larger the power coefficient, the stronger the weight of the nearby points. A cross-validation method based on jack-knifing resampling was conducted to quantify the accuracy of interpolated surfaces and compute 95% confidence intervals (CI). The predictive performance of models was summarised using the root-mean of squared residuals (RMSE) from the errors.

Patterns of malaria incidence and vector densities across the study village, unsurprisingly, showed large similarities. Higher malaria risk was observed in the eastern sub-villages, where hotspots were observed in Mage, the sub-village closest to the lake and of lower socioeconomic status. The maps demonstrated the presence of “hotspots” in households that lie on the edges of sub-villages and also “coldspots” in Sille and Ganjule. With limited resources available, the identification of hot- and cold-spots could inform policy makers and control programmes for targeted vector control. Nevertheless, further research is required to investigate why some areas are hot- and cold-spots.


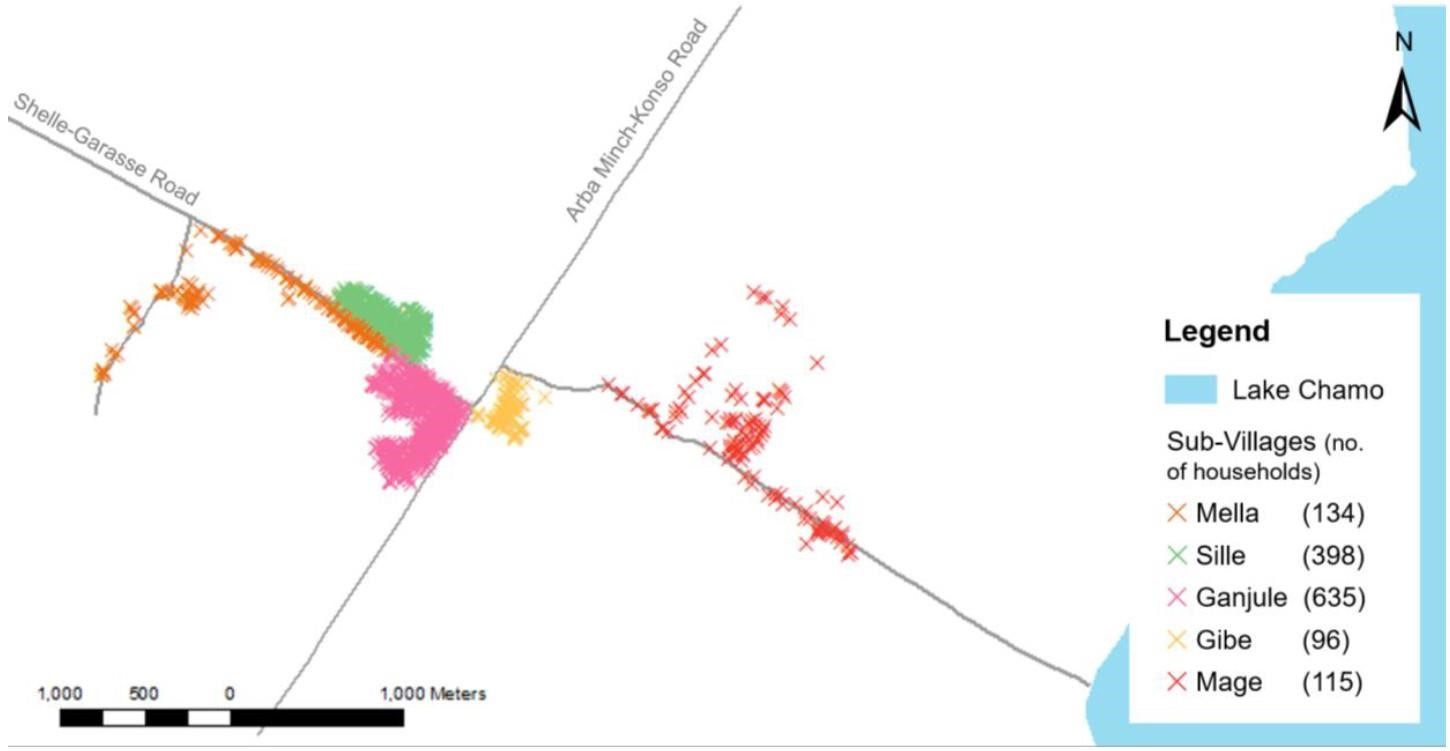


**Figure S3**. Map of all the 1378 households in Shelle Mella, divided into 5 sub-villages.


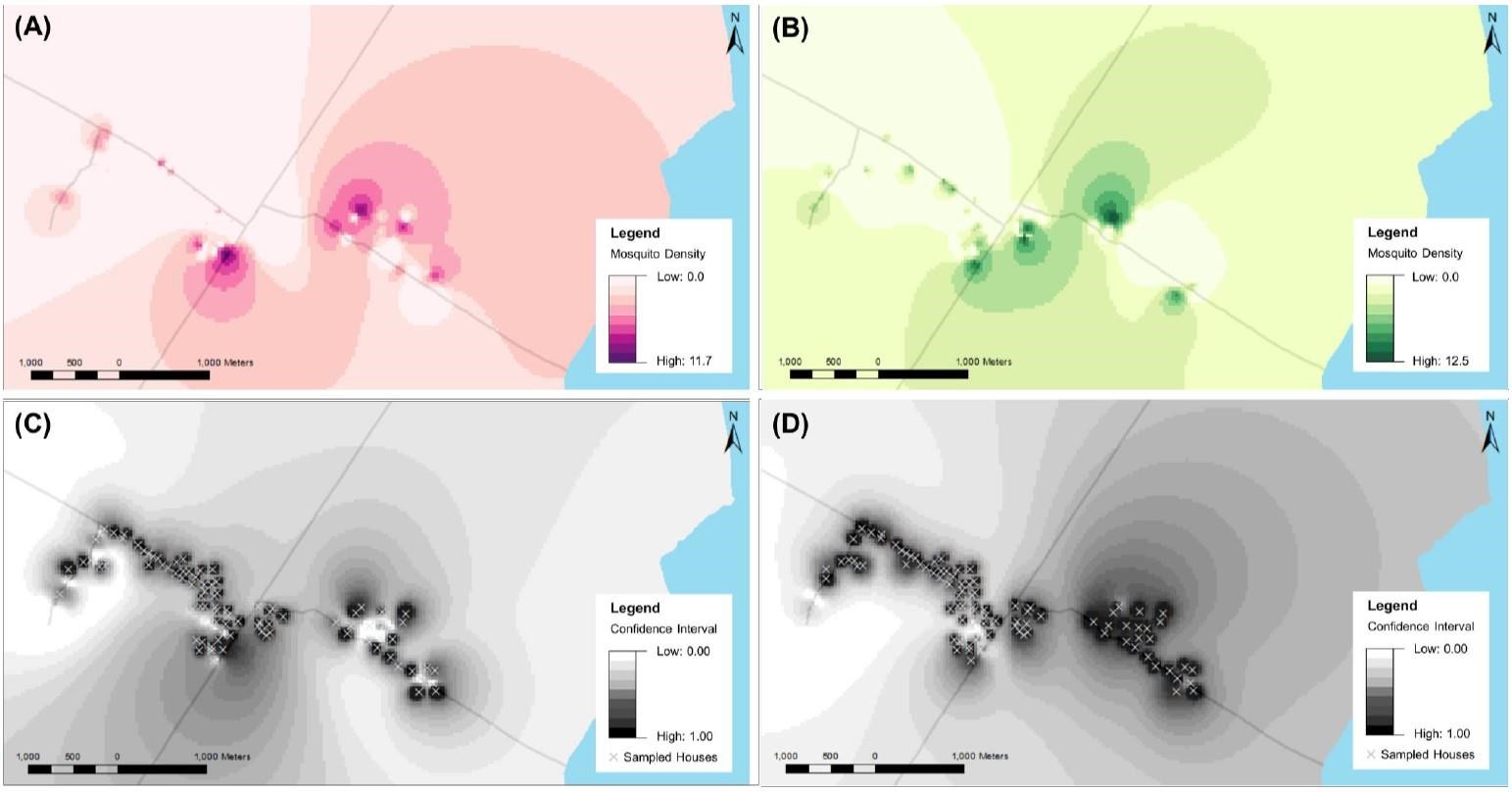


**Figure S4.** The predicted number of **(A)** mosquitoes found indoors and **(B)** outdoors per household across Shelle Mella, accompanied by the confidence intervals of the estimated numbers of **(C)** mosquitoes collected indoors and **(D)** mosquitoes collected outdoors as estimates of the accuracy of the interpolations.

**Table S2**. RMSE, a measure of differences between values predicted by the interpolated and the observed values, of malaria incidence and mosquito density, obtained through cross validation.

|  | RMSE  (null model) | RMSE  (mean of cross-validation) | Variance |
| --- | --- | --- | --- |
| Malaria prevalence | 0.903 | 0.904 | -0.001 |
| Mosquito density (total) | 3.122 | 2.663 | 0.147 |
| Mosquito density (indoors) | 2.441 | 2.189 | 0.103 |
| Mosquito density (outdoors) | 1.784 | 1.802 | -0.010 |

**Table S3**. Factors associated with malaria incidence (n=463).

| **Risk factor** | **N** | **Malaria cases^a^**  **(%)** | **Unadjusted univariate analysis** | | | **Adjusted multivariate analysis** | | |
| --- | --- | --- | --- | --- | --- | --- | --- | --- |
|  |  |  | OR | 95% CI | p-value | aORb | 95% CI | p-value |
| **Individual-level** | | |  | | |  | | |
| **Sex** | | |  | | |  | | |
| Male | 252 | 24 (9.5) | 1.00 | - | - | 1.00 | - | - |
| Female | 211 | 25 (11.8) | 1.46 | 0.76 – 2.81 | 0.255 | 1.59 | 0.81 – 3.12 | 0.177 |
| **Age** | | |  | | |  | | |
| 0-5 | 72 | 12 (16.7) | 2.76 | 1.14 – 6.69 | 0.025 | 2.88 | 1.17 – 7.11 | 0.022 |
| 6-15 | 135 | 15 (11.1) | 1.31 | 0.59 – 2.88 | 0.504 | 1.38 | 0.62 – 3.07 | 0.424 |
| 16-50 | 236 | 20 (8.5) | 1.00 | - | - | 1.00 | - | - |
| 51+ | 20 | 2 (10.0) | 1.25 | 0.23 – 6.85 | 0.795 | 1.11 | 0.20 – 6.28 | 0.903 |
| **Household level** | | |  | | |  | | |
| **Household size** |  |  |  |  |  |  |  |  |
| 1-3 | 41 | 5 (12.2) | 1.00 | - | - | 1.00 | - | - |
| 4-6 | 244 | 31 (12.7) | 1.01 | 0.30 – 3.45 | 0.985 | 0.94 | 0.26 – 3.38 | 0.926 |
| 7+ | 178 | 13 (7.3) | 0.54 | 0.14 – 2.08 | 0.374 | 0.54 | 0.12 – 2.43 | 0.424 |
| **Socioeconomic status** | | |  | | |  | | |
| 1^st^ quantile | 83 | 11 (13.3) | 1.00 | - | - | 1.00 | - | - |
| 2^nd^ quantile | 106 | 9 (8.5) | 0.63 | 0.20 – 2.02 | 0.435 | 0.64 | 0.19 – 6.28 | 0.475 |
| 3^rd^ quantile | 124 | 19 (15.3) | 1.20 | 0.41 – 3.48 | 0.737 | 1.39 | 0.44 – 4.36 | 0.575 |
| 4^th^ quantile | 150 | 10 (6.7) | 0.43 | 0.14 – 1.36 | 0.152 | 0.48 | 0.14 – 1.61 | 0.232 |
| **IRS** |  |  |  |  |  |  |  |  |
| No | 78 | 12 (15.4) | 1.00 | - | - | 1.00 | - | - |
| Yes | 385 | 37 (9.6) | 0.71 | 0.26 – 1.95 | 0.507 | 0.77 | 0.27 – 2.22 | 0.631 |
| **Number of nets** | | |  | | |  | | |
| 0 | 207 | 27 (13.0) | 1.00 | - | - | 1.00 | - | - |
| 1 | 113 | 11 (9.7) | 0.71 | 0.27 – 1.89 | 0.498 | 0.66 | 0.24 – 1.82 | 0.420 |
| 2+ | 143 | 11 (7.7) | 0.59 | 0.22 – 1.54 | 0.279 | 0.68 | 0.24 – 1.90 | 0.463 |
| **Number of cattle** | | |  | | |  | | |
| 0 | 93 | 10 (10.8) | 1.00 | - | - | 1.00 | - | - |
| 1-4 | 214 | 23 (10.7) | 0.97 | 0.34 – 2.70 | 0.946 | 0.89 | 0.30 – 2.62 | 0.837 |
| 5+ | 156 | 16 (10.3) | 0.83 | 0.26 – 2.58 | 0.744 | 1.06 | 0.28 – 3.97 | 0.932 |
| **Cattle to human ratio (CHR)** | | |  | | |  | | |
| 0 | 93 | 10 (10.8) | 1.00 | - | - | 1.00 | - | - |
| 10-50 | 143 | 16 (11.2) | 1.05 | 0.35 – 3.15 | 0.928 | 1.03 | 0.32 – 3.30 | 0.954 |
| 51-100 | 135 | 17 (12.6) | 1.05 | 0.35 – 3.21 | 0.927 | 1.04 | 0.31 – 3.49 | 0.952 |
| 101-450 | 92 | 6 (6.5) | 0.55 | 0.15 – 2.06 | 0.374 | 0.59 | 0.14 – 2.48 | 0.473 |
| **Cattle-keeping practice (CKP)** | | |  | | |  | | |
| No cattle | 123 | 10 (8.1) | 1.00 | - | - | 1.00 | - | - |
| Cattle sheds | 81 | 8 (9.9) | 1.20 | 0.34 – 4.27 | 0.775 | 1.04 | 0.27 – 4.00 | 0.959 |
| Cattle within compound | 259 | 31 (12.0) | 1.50 | 0.57 – 3.93 | 0.409 | 1.45 | 0.52 – 4.00 | 0.476 |
| **Environmental** | | |  | | |  | | |
| **Distance from wetland areas** | | |  | | |  | | |
| Close | 132 | 19 (14.4) | 1.00 | - | - | 1.00 | - | - |
| Medium | 179 | 16 (8.9) | 0.59 | 0.22 – 1.53 | 0.276 | 0.72 | 0.25 – 2.12 | 0.554 |
| Far | 152 | 14 (9.2) | 0.57 | 0.21 – 1.57 | 0.280 | 0.62 | 0.20 – 1.87 | 0.393 |
| **Distance from neighbouring lake, Lake Chamo** | | | | |  |  |  |  |
| Close | 48 | 2 (4.2) | 1.00 | - | - | 1.00 | - | - |
| Medium | 243 | 27 (11.1) | 3.29 | 0.57 – 18.91 | 0.182 | 2.57 | 0.41 – 16.13 | 0.313 |
| Far | 172 | 20 (11.6) | 3.02 | 0.51 – 17.89 | 0.223 | 2.05 | 0.31 – 13.57 | 0.456 |
| **NDVI** | | | | |  |  |  |  |
| Low | 74 | 6 (8.1) | 1.00 | - | - | 1.00 | - | - |
| Medium | 184 | 16 (8.7) | 1.14 | 0.33 – 3.91 | 0.834 | 1.25 | 0.34 – 4.63 | 0.736 |
| High | 205 | 27 (13.2) | 1.66 | 0.51 – 5.46 | 0.401 | 1.66 | 0.48 – 5.74 | 0.424 |
| **Cattle density** | | | | |  |  |  |  |
| Low | 253 | 33 (13.0) | 1.00 | - | - | 1.00 | - | - |
| Medium | 164 | 15 (9.2) | 0.70 | 0.30 – 1.62 | 0.400 | 0.60 | 0.24 – 1.54 | 0.290 |
| High | 46 | 1 (2.2) | 0.13 | 0.01 – 1.25 | 0.077 | 0.14 | 0.01 – 1.35 | 0.089 |
| **People density** | | | | |  |  |  |  |
| Low | 314 | 37 (11.8) | 1.00 | - | - | 1.00 | - | - |
| Medium | 105 | 9 (8.6) | 0.68 | 0.25 – 1.84 | 0.446 | 0.70 | 0.23 – 2.12 | 0.534 |
| High | 44 | 3 (6.8) | 0.54 | 0.12 – 2.53 | 0.436 | 0.48 | 0.10 – 2.36 | 0.364 |

*OR* odds ratio ^a^ Self-reported malaria incidence ^b^ Adjusted for age, sex and socioeconomic status

**Table S4.** Summary of the designs and main outcomes of previous zooprophylaxis-related studies performed in Ethiopia.

| **Reference** | **Study design** | **Outcome(s)** | **Findings** |
| --- | --- | --- | --- |
| Ghebreyesus  *et al.* (2000) [1] | Questionnaire to document individual and household characteristics | Malaria incidence of children <10 years | - Zoopotentiation in mixed dwellings: animals sleeping inside houses had significant associations with malaria incidence (RR = 1.92) |
| Seyoum *et al.* (2002) [2] | Mosquito collections via CDC light traps and human landing catches (HLCs) at 3 sites:   - Mixed human-cattle dwelling - Separate human-cattle dwelling (cattle shed) - Human-only dwelling | Malaria incidence of children <10 years | - Zoopotentiation in mixed dwellings: higher *An. arabiensis* human biting rate (HBR) in the presence of cattle or goats *vs* without livestock (3.50, 3.38 *vs* 1.43) - No effect in mixed dwellings: no clear *An. pharoensis* HBR pattern - Zooprophylaxis in separate human-cattle dwellings |
| Habtewold *et al.* (2001) [3] | Mosquitoes collected from 3 different sites:   - A: Separate human-cattle dwelling (cattle shed) - B: Mixed human-cattle dwelling - C: Separate human-cattle dwelling (humans on elevated platforms) | Blood meal analysis and sporozoite rate | - Zooprophylaxis in separate human-cattle dwellings: proportion of mosquitoes that fed on livestock significantly higher than on humans in site C - No effect: proportion of mosquitoes that fed on humans and livestock did not differ significantly in sites A or B |
| Yewhalaw *et al.* (2014) [4] | Mosquito collections via:   - Indoor resting collections (IRC) and CDC light traps - Outdoor resting collections (ORC) from all potential natural resting sites | Blood meal analysis and sporozoite rate | - Zooprophylaxis: higher proportion of bovine and ovine blood than human |
| Hadis *et al.* (1997) [5] | Mosquitoes collected from:   - Human dwellings - Cattle sheds - Mixed dwellings | Blood meal analysis | - Zooprophylaxis in mixed dwelling: proportion of bovine blood was three times higher than that of human blood |
| Massebo *et al.* (2013) [6] | Mosquito collections via:   - IRC and CDC light traps - ORC from pit shelters | Blood meal analysis | - Zooprophylaxis: higher bovine blood index (69%) than human blood index (44%) in *An. arabiensis* |
| Massebo *et al.* (2015) [7] | Mosquito collections via:   - IRC and CDC light traps - ORC from pit shelters | Blood meal analysis | - Zooprophylaxis: *An. arabiensis* showed strong preference for bovine meal over human (despite three-fold higher prevalence of humans) |
| Habtewold *et al.* (2004) [8] | Mosquitoes collected from HLCs next to:   - Deltamethrin-treated cattle - Untreated cattle | No. of mosquitoes caught in different conditions / traps | - No effect against *An. arabiensis*: HBR did not differ in the presence of treated or untreated ox - Zooprophylaxis against *An. pharoensis*: HBR reduced by 50% in presence of treated or untreated ox |

| Tirados *et al.* (2006) [9] | Mosquito collections via:   - IRC and CDC light traps - ORC from pit shelters & cattle-baited traps (CBT) - Odour-baited entry traps | No. of mosquitoes caught in different conditions / traps & sporozoite rate | - Zoopotentiation: more mosquitoes caught using HLCs than CBTs |
| --- | --- | --- | --- |
| Tirados *et al.* (2011) [10] | - Experiment 1: HLC outdoors with a ring of cattle - Experiment 2: human-baited trap and CBT with or without a ring of cattle - Experiment 3: HLC indoors or outdoors - Experiment 4: HLC indoors with or without ring of cattle outdoors | No. of mosquitoes caught in different conditions / traps | - Zooprophylaxis: indoor catch of *An. arabiensis* significantly reduced by 49% with cattle ring (exp. 4) - No effect: on *An. arabiensis* when humans are outdoors (exp. 1) - Zooprophylaxis: on *An. pharoensis* when humans are indoors and outdoors (exp. 1) |

# References

1. Ghebreyesus TA, Haile M, Witten KH, Getachew A, Yohannes M, Lindsay SW, et al. Household risk factors for malaria among children in the Ethiopian highlands. Trans R Soc Trop Med Hyg. 2000;94:17–21.
2. Seyoum A, Balcha F, Balkew M, Ali A, Gebre-Michael T. Impact of cattle keeping on human biting rate of anopheline mosquitoes and malaria transmission around Ziway, Ethiopia. East Afr Med J. 2002;79:485–90.
3. Habtewold T, Walker AR, Curtis CF, Osir EO, Thapa N. The feeding behaviour and *Plasmodium* infection of *Anopheles* mosquitoes in southern Ethiopia in relation to use of insecticide-treated livestock for malaria control. Trans R Soc Trop Med Hyg. 2001;95:584–6.
4. Yewhalaw D, Kelel M, Getu E, Temam S, Wessel G. Blood meal sources and sporozoite rates of Anophelines in Gilgel-Gibe dam area , Southwestern Ethiopia Afr J Vector Biol. 2014;89:34.
5. Hadis M, Lulu M, Makonnen Y, Asfaw T. Host choice by indoor-resting *Anopheles arabiensis* in Ethiopia. Trans R Soc Trop Med Hyg. 1997;91:376–8.
6. Massebo F, Balkew M, Gebre-Michael T, Lindtjørn B. Blood meal origins and insecticide susceptibility of *Anopheles arabiensis* from Chano in South-West Ethiopia. Parasit Vectors. 2013;6:1–10.
7. Massebo F, Balkew M, Gebre-Michael T, Lindtjørn B. Zoophagic behaviour of anopheline mosquitoes in southwest Ethiopia: Opportunity for malaria vector control. Parasit Vectors. 2015;8:645.
8. Habtewold T, Prior A, Torr SJ, Gibson G. Could insecticide-treated cattle reduce Afrotropical malaria transmission? Effects of deltamethrin-treated Zebu on *Anopheles arabiensis* behaviour and survival in Ethiopia. Med Vet Entomol. 2004;18:408– 17.
9. Tirados I, Costantini C, Gibson G, Torr SJ. Blood-feeding behaviour of the malarial mosquito *Anopheles arabiensis*: Implications for vector control. Med Vet Entomol. 2006;20:425–37.
10. Tirados I, Gibson G, Young S, Torr SJ. Are herders protected by their herds? An experimental analysis of zooprophylaxis against the malaria vector *Anopheles arabiensis*. Malar J. 2011;10:1–8.
